# Aβ40 Aggregation under Changeable Conditions

**DOI:** 10.3390/ijms24098408

**Published:** 2023-05-07

**Authors:** Jofre Seira Curto, Maria Rosario Fernandez, Josep Cladera, Núria Benseny-Cases, Natalia Sanchez de Groot

**Affiliations:** 1 Unitat de Bioquímica, Departament de Bioquímica i Biologia Molecular, Universitat Autònoma de Barcelona, 08193 Barcelona, Spain; jofre.seira@uab.cat (J.S.C.); rosario.fernandez@uab.cat (M.R.F.); 2 Unitat de Biofísica, Departament de Bioquímica i Biologia Molecular, Centre d’Estudis en Biofísica, Universitat Autònoma de Barcelona, 08193 Barcelona, Spain; josep.cladera@uab.cat

**Keywords:** amyloid-β-peptide, charge repulsion, aggregation

## Abstract

Homeostasis is crucial for cell function, and disturbances in homeostasis can lead to health disorders. Under normal conditions, intracellular pH is maintained between 7.35 and 7.45. Altered endosomal and lysosomal pH together with a general drop in brain pH are associated with the aggregation of amyloid-β-peptide (Aβ) and the development of Alzheimer’s disease. Under acidic conditions, close to the Aβ isoelectric point, the absence of charges favors the formation of intermolecular contacts and promotes aggregation. Here, we analyzed how pH levels affect the aggregation of Aβ40 considering the variations in brain pH and the coexistence of different aggregated conformations. Our results suggest that different macromolecular conformations can interact with each other and influence the aggregation process. In addition, we showed that neutral pH and physiological salt concentrations favor a slow aggregation, resulting in ordered, stable fibrils, with low cytotoxic effects. Overall, we highlight the complexity of the aggregation processes occurring in different physiological and pathological environments.

## 1. Introduction

The intracellular space is complex and dynamic. Cellular homeostasis depends on the maintenance and regulation of internal conditions in time and space. Impaired homeostasis is an age-related problem that has been observed in many neurodegenerative diseases. In these disorders, impaired removal of misfolded and aggregated proteins can trigger a cascade of events that ultimately leads to cell death [1,2,3].

**Figure 1:**
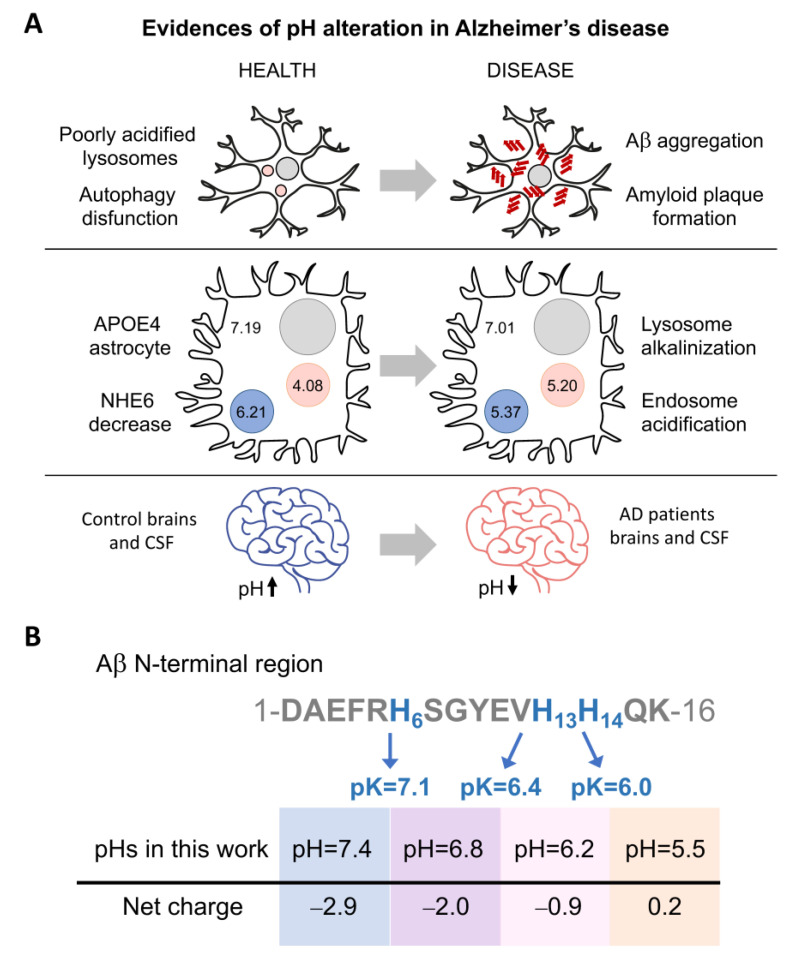
pH influences the net charge of Aβ and Alzheimer’s disease development. (**A**) Three pieces of evidence of pH alterations in Alzheimer’s disease. (i) Poor acidification of autolysosomes results in autophagy dysfunction and precedes Aβ deposition and amyloid plaque formation [2]. (ii) In ApoE4 astrocytes (with the E4 allele of apolipoprotein E), downregulation of the Na+/H+ exchanger NHE6 results in over-acidification of the endosomes and pH increase in lysosomes [6]. (iii) Lower pH has been measured in the brains and cerebrospinal fluid (CSF) of patients with Alzheimer´s disease (AD) [5]. (**B**) Scheme of the Aβ N-terminal sequence, comprising histidines whose protonation is affected in the pH range analyzed in this study. The scheme shows the amino acid position and pK of the histidines and the net charge at each pH.

Similarly, the maintenance of an appropriate acid–base balance is essential for the correct functioning of human body processes [4]. Under normal physiological conditions, the intracellular and interstitial pH is between 7.35 and 7.45. Changes in pH can inhibit enzymatic activity, interrupt metabolic pathways, and disturb the membrane transport of proteins. Significantly lower pH has been measured in the brain and cerebrospinal fluid (CSF) of patients with Alzheimer’s disease (AD) [5] (Figure 1A). In addition, alteration of endosomal and lysosomal pH has been related with problems in amyloid-β-peptide (Aβ) clearance and the formation of extracellular Aβ plaques [2,6].

Aβ is generated through the proteolytic cleavage of α-, β-, and γ-secretases on amyloid precursor protein (APP), a single-pass transmembrane protein [7,8,9]. This cleavage can produce different extracellular Aβ peptides with lengths ranging from 37 to 49; however, the most abundant forms are Aβ40 (~90%) and Aβ42 (~10%) [10,11,12]. In a disease context, Aβ42 is the main major constituent of the amyloid plaques in the brains of patients with Alzheimer’s disease, whereas Aβ40 aggregates accumulate at the arteriolar walls in cerebral amyloid angiopathy [12,13,14]. The two extra residues at the C-terminus Aβ42 result in faster aggregation kinetics compared to Aβ40. Interestingly, under mixed conditions, both peptides can interact and influence each other’s aggregation [15,16,17,18]. Aβ can enter the cell via endocytosis for subsequent degradation by lysosomes [2,6,19]. When cell homeostasis is affected, this degradation pathway can fail, causing Aβ accumulation and aggregation. Additionally, the acidity of these organelles can accelerate the aggregation process (Figure 1A) [2,6,20].

Protein aggregation is highly influenced by hydrophobicity and net charge [4,21,22,23,24]. When the pH approaches the isoelectric point (pI), the absence of charges removes the repulsion forces and favors the formation of intermolecular contacts involved in the aggregation process. The presence of salt ions can have a similar effect by shielding repulsive ionic interactions [25]. Our previous studies with Aβ40 showed that the aggregates formed at neutral or acidic pH have different structures and cell toxicities [23,24]. However, the processes inside the cell are dynamic, and pH can change regularly in organelles [4,6]. Based on this, we aim to go forward here and shed light on the different toxic protein species that can arise under moderate pH and salt concentration changes. For this purpose, we analyzed how pH levels affect the aggregation of Aβ40 considering the changeable conditions of the intracellular space and the pH alterations that can occur locally in the extracellular space. Based on the pH reported for the altered endosomes and lysosomes in AD (Figure 1B), we focused on a range of pH from 5.5 to 7.4 and salt from 0 to 150 mM of NaCl (the most abundant salt found in multicellular organisms [25,26,27] widely used in aggregation assays [25,26,28,29]). We characterized the biophysical differences between the aggregates that form under these conditions considering the possible coexistence of different aggregated conformations and their ability to interact and interconvert. We found that salt and pH modulate the structure, stability, and cellular toxicity of the aggregates. Overall, we propose that even small cellular changes in pH and salt concentration could result in a complex and variable mixture of multimeric forms, which are able to interfere with each other to different degrees.

## 2. Results

### 2.1. pH and Salt Effect on Aβ40 Aggregation

Taking into consideration the different intracellular environments where the presence of Aβ40 has been detected [6], and with the aim of understanding how pH and NaCl concentration may influence the kinetics and conformation of Aβ40 aggregates, we measured amyloid aggregation at different pH and NaCl conditions. Here, we specifically tested four different pH values (7.4, 6.8, 6.2, and 5.5) (Figure 1B), similar to those previously measured in endosomes and lysosomes (Figure 1A), and seven concentrations of NaCl ranging from 0 to physiological conditions (150 mM), to understand the extent to which the presence of this salt influences the aggregation of Aβ40 at different pH values.

**Figure 2:**
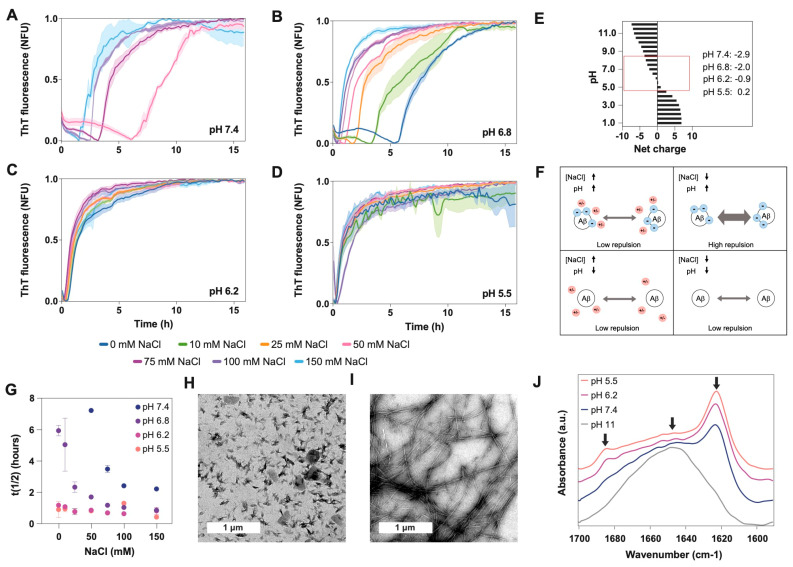
Effect of pH on Aβ40 aggregation and conformation. We measured aggregation kinetics at four different pH values: (**A**) 7.4, (**B**) 6.8, (**C**) 6.2, and (**D**) 5.5. For each pH, we also analyzed seven NaCl concentrations ranging from 0 to 150 mM. (**E**) Plot showing the Aβ40 net charge at different pH values, calculated with Protein Calculator v3.3 (Chris Putnam, Scripps Research Institute). (**F**) Scheme showing the repulsive forces between Aβ molecules at high and low pH and high and low NaCl concentrations. (**G**) Plot showing the relationship between the half-life (t(1/2)) of the kinetics presented in panels (**A**–**D**) and the NaCl concentration. TEM images of aggregates formed at (**H**) pH 5.5 and (**I**) pH 7.4. (**J**) FTIR spectra of Aβ40 aggregates incubated at four different pH values (5, 6, 7.4, and 10) and for 24 h. Arrows indicate the position of the following wavelengths: 1690 cm^−1^, 1645 cm^−1^ and 1623 cm^−1^. The spectrum at pH 10 demonstrates that the initial stock solution does not contain beta-sheet structure. It just shows a wide band at 1645 cm^−1^ corresponding to random structures, indicating that the peptide is monomeric at the beginning of the experiments. The peaks at 1690 cm^−1^ and 1623 cm^−1^ indicate the presence of antiparallel beta-sheet structures.

Aβ40 aggregation was monitored every 10 min under quiescent conditions at 37 °C (Figure 2A–D and Figure S1). In general, lag time and half-life (t1/2) decrease when the pH becomes more acidic, consistent with the charge reduction (from −3 to nearly 0) when approaching the isoelectric point (pI, 5.3) (Figure 2E,F and Figure S2). The increase in salt has a similar effect, which can be explained by the reduction of repulsive forces exerted by the peptide’s negative charges (Figure 2E,F). However, at a pH close to the pI, the repulsion effect was lower, we did not observe any effect of NaCl on the aggregation process, and the half-lives were similar for all conditions (Figure 2D). In contrast, when the net charge was −3 (pH 7.4) and the salt concentration was low (from 0 to 25 mM), the aggregation kinetics were too slow to be measured over the time scale reported here (16 h) (Figure 2A). Kinetic analysis for longer times showed that they achieved a plateau and remained stable (Figure S3). Meisl and coworkers obtained similar results when analyzing the effect of ionic strength on Aβ42 aggregation [25]. They found that the reduction in electrostatic repulsion surrounding the peptide can accelerate the incorporation of new monomers into the fibrils, which in turn reduces the impact that the fragmentation can have on the elongation speed [25].

To study the aggregates’ conformation, we chose a 100 mM NaCl concentration, which is close to the physiological values, and two extreme pHs (7.4 vs. 5.5). We analyzed the morphology of the aggregates using transmission electron microscopy (TEM) (Figure 2F,G) and the secondary conformation using infrared spectroscopy (IR) (Figure 2H and Figure S4). Under these experimental conditions, the aggregates at pH 7.4 are arranged into long and straight fibrillar structures that tend to group into clumps. However, at pH 5.5 the aggregates are shorter, less ordered, and more dispersed. This agrees with the IR absorbance spectra, which show the presence of oligomeric/amorphous aggregates at pH 5.5 and, in a lower amount, at pH 6.2. The IR spectra at these pH values show a characteristic band at 1690 cm^−1^ together with a clear beta-sheet band at 1623 cm^−1^, which is associated with the presence of an antiparallel β-sheet, which is typical of oligomers and amorphous aggregates. Several studies have associated the presence of bands at 1623 cm^−1^ and 1690 cm^−1^ to antiparallel beta, whereas the sole presence of the band around 1620 cm^−1^ is associated with parallel (fibrillar) beta-sheet structures [30,31]. Consistently, at pH 7.4, we detected only the 1623 cm^−1^ absorption peak characteristic of the beta-sheet structure, suggesting the presence of fibrils (Figure 2H and Figure S4). Conversely, at a net charge above -5 (pH 11), no signal of β-sheet structure (1623 cm^−1^) or amorphous aggregates (1690 cm^−1^) was detected, just a wide peak at 1645 cm^−1^ typical of an unfolded monomeric form [32]. In agreement with this, the initial stock sample at pH 11 only showed monomeric particles under dynamic light scattering (DLS) [33,34], and no aggregated structures were observed at the TEM grid (Figure S5).

### 2.2. pH Exchange and Cross-Seeding

**Figure 3:**
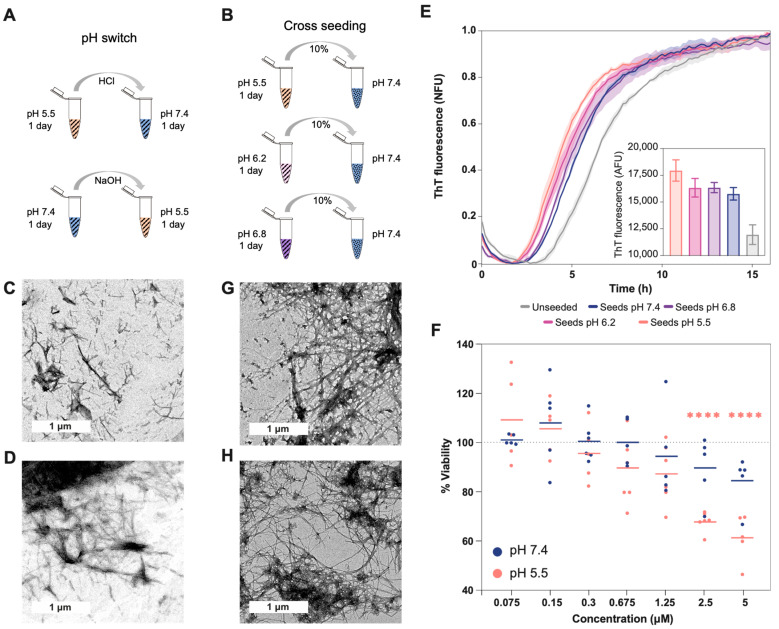
Effect of cross-seeding and pH switch on Aβ40 aggregation and conformation. (**A**) Scheme showing the different steps of the pH switch assay (related to images (**C**,**D**)). (**B**) Scheme showing the different steps of the cross-seeding assay (related to images (**E**,**G**,**H**)). The striped pattern indicates that Aβ40 is aggregated, and the dotted pattern that Aβ40 is soluble. TEM images for the aggregates formed: (**C**) with the first 24 h at pH 5.5 and the second ones at 7.4, (**D**) with the first 24 h at pH 7.4 and the second ones at 5.5, (**G**) at pH 7.4 with seeds formed at pH 5.5, and (**H**) at pH 5.5 with seeds formed at pH 7.4. (**E**) Aggregation kinetics at pH 7.4 with and without different seed types. The fluorescence measured with seeds formed at pH 5.5 is significatively larger than with the seeds formed at pH 7.4 (*p*-value < 0.01). The inside plot shows no normalized fluorescence after 16 h of aggregation. The t1/2 in hours for the different samples are as follows: unseeded (6.61 ± 0.10), seeds pH 7.4 (5.38 ± 0.01), seeds pH 6.8 (5.1 ± 0.259), seeds pH 6.2 (4.83 ± 0.17), and seeds pH 5.5 (4.44 ± 0.19). (**F**) The percentage of cell viability after incubation with different concentrations of Aβ40 aggregated at different pH values (measured using the MTT assay). The plot shows triplicates of the two independent assays. The lines represent the mean corresponding to each concentration of the added aggregates. The significance against the control of 100% viability was measured using the Bonferroni test (****, <0.0001). At concentrations of 2.5 uM and 5 uM of aggregates, the fluorescence difference between seeds formed at pH 5.5 and seeds formed at pH 7.4 is also significant (*p* = 0.001 and *p* = 0.0003, respectively). The measurements of the different controls are shown in Figure S6.

To understand how pH changes can affect the structure of preformed aggregates and the interaction between them, we analyzed (i) the effect of pH change (Figure 3A) and (ii) the cross-seeding between aggregates formed at different pH values (Figure 3B). The TEM images show that the aggregates grown in acidic conditions are pH sensitive, and after 24 h of increasing the pH to neutral, the aggregates progressed to straight fibrils, which were shorter than those grown at pH 7.4. These fibrils were also more abundant, to a similar extent as the amorphous aggregates at acidic pH (Figure 3C). Meanwhile, when the pH of the aggregates grown at 7.4 was decreased, no significant structural changes were observed (Figure 3D). 

Based on the observed morphological changes and the labile character of the aggregates formed at acidic pH, we analyzed their ability to seed the aggregation at pH 7.4 (Figure 3E). This is important because fibril fragility is associated with the presence of shorter aggregates, the release of a larger number of growing ends, and the acceleration of the aggregation process [22,35]. Accordingly, we measured shorter half-life times (t1/2) when Aβ40 was seeded with aggregates formed at a low pH, indicating a higher seeding ability. Interestingly, the TEM images from the different seeding assays showed aggregates with similar morphology: mature fibrils that, in some cases, formed clumps (Figure 3G,H).

### 2.3. AΒ40 Aggregates Toxicity

Less stable aggregates are associated with the formation of more endpoints and higher cellular damage [22,35]. Along these lines, we measured cell viability, using the MTT assay, after 24 h of incubation with Aβ40 aggregated at pH 5.5 and 7.4 (Figure 3F and Figure S6). The colorimetric measures showed a significant decrease in viability at concentrations above 1.25 uM, but only in cells incubated with aggregates formed at pH 5.5 when the viability decreased to close to 40%. Importantly, we previously demonstrated, using μFTIR, that cell culture conditions do not affect the presence of Aβ40 amyloid fibrils formed at pH 7.4 or pH 5.5 [36]. In addition, this assay showed an increased level of oxidation in cells incubated with aggregates formed at pH 5.5 [36], in agreement with the present results.

## 3. Discussion

### 3.1. pH Effect on Aβ40 Aggregation

Recent studies have recapitulated the intracellular origin of the Aβ aggregates [1,2,3,6,19,37], which are related to autophagy impairment and pH imbalances. Lee and co-workers observed, in AD mouse models, that the poor acidification of autolysosomes results in autophagy dysfunction and precedes Aβ deposition and amyloid plaque formation [2]. In the case of the E4 allele of apolipoprotein E (ApoE4, the strongest genetic factor in sporadic AD), downregulation of the Na^+^/H^+^ exchanger NHE6 results in over-acidification of endosomes and inhibition of Aβ clearance [6]. In this context, in ApoE4 astrocytes, the pH of the endosome was reduced from 6.21 to 5.37, and the pH of the lysosome increased from 4.08 to 5.20.

However, pH alterations were not only detected in these intracellular organelles [11,19,29,38,39,40,41,42]. The postmortem brains and cerebrospinal fluid of AD patients also show a significantly lower, although moderate, pH compared to the controls. Infusion of low-pH cerebrospinal fluid, in APP-PS1 mice, increases Aβ plaque load [4]. Moreover, a pH reduction can also be found under inflammatory and apoptotic processes and at the surface of anionic phospholipid membranes [5,43]. Overall, these events could place Aβ close to pI, reducing its solubility and favoring its self-association and aggregation [4,21,22].

As the pH changes from physiological (7.4) to the pI (5.3) [44], His6, His13, and His14 are protonated successively, generating new interactions and favoring the transition from soluble to aggregated [20,26]. Acidity affects the morphology and toxicity of Aβ aggregates (Figure 2 and Figure 3). Protein aggregation is a very sensitive process, and the interaction and rearrangement of the molecules during the aggregate assembly could be influenced by multiple factors (temperature, charges, hydrophobicity, ionic strength), resulting in different macromolecular conformations or strains [21,22,23,24,36,38,41,45]. Amorphous and less organized structures are also associated with lower stability, large reactivity, and cellular toxicity [21,22,23,24,36,39,41,45]. Thus, small changes in the Aβ uptake and degradation could affect the self-assembly process [1,2,3,6,37,46,47]. Moreover, under changeable conditions, different conformational assemblies can coexist, interact, and influence each other’s aggregation.

### 3.2. Effect of pH Exchange and Cross-Seeding on Aβ40 Aggregation

Here, we evaluated the effect of changes in the electrostatic repulsion on Aβ40 aggregation, taking into consideration the different environments that this peptide can encounter in the cell [1,6,22,37,38,39,48,49]. We also presumed that the conditions in the cell can change and that different Aβ40 assemblies can rise and interact. Based on this assumption, we performed a series of experiments, ranging from neutral (7.4) to acidic pH (5.5) and from physiological concentrations to 0 uM of NaCl. We also analyzed the effect of pH alteration, decreasing the pH from neutral or increasing it from an acidic solution. Following these alterations, we analyzed the coexistence of assemblies grown under different conditions. 

**Figure 4:**
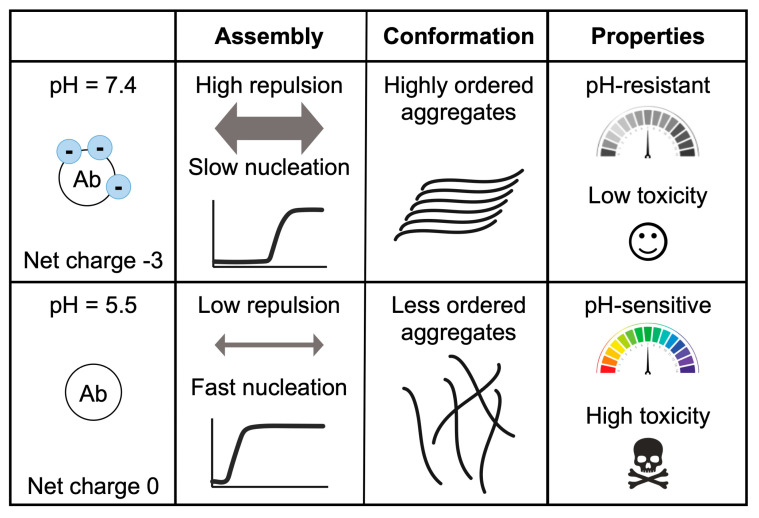
Effect of pH on Aβ40 aggregation, conformation, and toxicity. This table summarizes the differences between the aggregates formed at pH 7.4 and pH 5.5. At these pH values, Aβ40 has a different net charge (−3 vs. 0), resulting in different repulsion forces and aggregation speeds. Meanwhile, at pH 7.4 the aggregates are ordered and pH resistant and exhibit low toxicity. At pH 5.5, the aggregates presented abundant oligomers and protofibrils, which are pH sensitive and toxic.

In agreement with previous publications, our experiments support electrostatic repulsion as a driving force in aggregation, modulated by the change of pH and salt concentration [23,24,25,26,38,41,50,51]. The pH decrease from 7.4 to 5.5 results in a net charge variation from -3 to 0.2, and by getting closer to Aβ40′s pI, the aggregation kinetics become faster (Figure 4). Similarly, the increase in NaCl reduces the repulsion between the negative charges, accelerating aggregation, except under acidic pH, when the charges are already nearly neutralized. The aggregates formed at different pH values have different morphologies and structural content. As previously reported, under neutral conditions, the aggregates are more fibrillar and contain fewer oligomeric and protofibril structures than under acidic pH conditions [23,24,51]. A faster aggregation process may hinder the correct assembly of the different molecules, resulting in less ordered aggregates than fibrils presenting antiparallel β-sheet structures, similar to those detected in vitro in the early stages of the amyloid fibril formation process [30,31]. Here, we altered the aggregation conditions by incubating the preformed aggregates at a second different pH or by seeding the process with different assemblies (Figure 3A,B). Overall, different aggregates were observed at different pH values. Aggregates grown under acidic conditions are pH sensitive and can seed aggregation stronger than those formed at neutral pH, resulting in a larger amount of aggregates (Figure 3E). This could be a consequence of the different arrangements of these assemblies. In general, less ordered forms, such as oligomers or protofibrils, are associated with lower stability and higher toxicity (such as the generation of reactive oxygen species or the disruption of membranes) [1,23,24,39,51,52,53]. In line with this, we have previously reported that, in leakage experiments, the aggregates formed at pH 5.5 have a higher capacity to disrupt phosphatidylcholine model membranes than fibrils formed at pH 7.4 [23].

In relation to the stability of the different aggregates, we observed that the aggregates formed at pH 7.4 barely changed their morphology at acidic pH, while those formed at pH 5.5 evolved to a more ordered form when placed at neutral pH (Figure 4). This suggests that aggregates formed at pH 7.4 may be more stable (less pH sensitive) than those grown at pH 5.5. In addition, the smaller size and higher abundance of aggregates formed at pH 5.5 may also provide more end-points for amyloid fibril growth [22].

### 3.3. Aβ40 Aggregation under Complex Conditions

Low pH can be found in intracellular organelles as well as in the presence of oxidized or pre-apoptotic membranes. These membranes present negative charges on their surfaces, which increase the concentration of protons and cause a local pH decrease [23]. In addition, pathological vascular events, such as ischemia and microhemorrhages related to AD, can cause a lack of oxygen, leading to a local decrease in pH in the extracellular space [54]. In patients with Alzheimer’s disease, there are changes in the concentrations of several ions. For example, Vitvitsky and co-workers found an increased concentration of positive ions, Na^+^ and K^+^, in comparison with control brains, pointing to a possible accelerated aggregation process [55].

Aβ’s pH dependence may also be associated with the formation of different conformational strains and with disease heterogeneity [21,39,56]. Accordingly, seeding experiments with Aβ aggregates obtained from the brains of patients with AD affected by different phenotypes resulted in structurally distinct fibrils [21,22,57]. In this way, oligomers have been reported to be less stable and more toxic than amyloid fibrils [1,23,24,39,51,52,53]. Phenotypic severity has been also associated with fibril fragility, because its fragmentation releases new growing ends that can seed new fibrils and accelerate the aggregation process [22,35]. Our results showed that the aggregates formed under acidic conditions present higher cytotoxicity and seeding. This agrees with our previous work where larger amounts of oligomers were present at lower pHs [23,24], but also suggests the presence of a larger number of endpoints. These results also point to an amplifying effect (Figure 3E); although aggregation is slow at neutral pH, the presence, at some point, of seeds formed at lower pHs could rapidly saturate the cell with highly stable aggregates and trigger the formation of extracellular plaques [2]. In fact, the study of AD mouse model APP/PS1 brains using FTIR microscopy has demonstrated the presence of non-fibrillar aggregates in situ, which is compatible with the FTIR spectra of oligomers and aggregates formed at low pH in the early stages of the disease (3–6 months). At later stages of the disease, a decrease in this type of aggregate was observed, and a higher number of fibrillary plaques were found [58]. The present work agrees with the co-existence of different macromolecular conformations and their possible cooperation triggering a harmful aggregation process [21]. In addition, it supports neutral pH and physiological salt concentrations being conditions that favor a slow aggregation process, resulting in ordered, stable, and less cytotoxic fibrils. This agrees with previous works showing that fibrils are safer aggregates than oligomers or protofibrils [22,35]. Based on these results, blocking or slowing down the aggregation process by favoring electrostatic repulsion could be an effective strategy to reduce toxic effects [2,6]. Moreover, owing to the sensitivity of aggregates to these variable conditions, it is possible to control the conformation of aggregates and their properties, a strategy already exploited by the cell to form functional amyloid structures [59]. In the case of Aβ, this could be a positive and evolutionarily selected property to activate its antimicrobial activity, since on the bacterial membranes the negative charges could favor Aβ’s aggregation and the consequent cell disruption [60]. Overall, the data presented here highlight the complexity of the aggregation processes occurring inside the cell and in the extracellular space and support the idea that the physio-pathological processes able to modify pH can play a significant role in triggering the onset of Aβ40 aggregation.

## 4. Material and Methods

### 4.1. AΒ40 Peptide Preparation

Synthetic Aβ40 (DAEFRHDSGYEVHHQKLVFFAEDVGSNKGAIIGLMVGGVV-NH_2_) was purchased from Proteogenix (Schiltigheim, France). Stock solutions were prepared by dissolving 1 mg of the peptide to a final concentration of 250 μM and adding 20 mM sodium phosphate buffer, 0.04% NH3, and NaOH to a final pH of 11. Then, the peptide was sonicated for ten minutes (Fisherbrand Pittsburgh, PA, USA, FB15051) without sweep mode and stored at −80 °C until needed [23].

All samples were prepared in low protein-binding microcentrifuge tubes (ThermoFisher Scientific, Waltham, MA, USA). The pH was measured at the start and end of all the aggregation reactions to confirm that it remained stable. For the pH change assays, 100 µL of sample after 24 h of aggregation at pH 7.4 was used, and 20 mM HCl was added to reach a pH of 5.5. For aggregates formed at pH 5.5, 20 mM NaOH was added to reach a pH of 7.4. For the seeding assays, 300 µL of preformed aggregates was obtained after 24 h of aggregation and 5 min of sonication. Preformed seeds were added to the corresponding aggregation reactions to a final seed concentration of 2.5 mM. The monomeric concentration of Aβ40 used was 25 µM. The control of unseeded reaction has the buffers at pH 7.4 and 25 µM of monomeric Aβ40 (everything but the seeds).

### 4.2. ThT Aggregation Kinetics

Thioflavin T was dissolved in Milli-Q water at 5 mM stock, filtered with a 0.2 μm filter, diluted to 0.5 mM, and stored at −20 °C prior to use. ThT fluorescence was measured every 10 min using a 440 nm excitation filter and a 480 nm emission filter using bottom-optics in a plate reader (TECAN Infinite+ NANO). Samples were placed in a flat-bottom, black, non-binding 96-well plate (Greiner bio-one). A total of 100 µL of sample was added per well. Each condition was measured in triplicate. The Aβ40 peptide stock, initially at pH 11, was diluted to 25 µM in sodium phosphate buffer under the corresponding pH and salt conditions. HCl was added at a concentration of 15 mM to reach a pH of 5.5. The pH in the wells was measured both at the start and end of the experiment to ensure the pH stability throughout the aggregation process. ThT was added to 20 mM final concentration. The aggregation reaction was performed at 37 °C, under quiescent conditions. The t1/2 is the time necessary, at a given condition, to reach 50% of the final fluorescence signal. The lag phase was calculated by considering its ending as the point at which it reached 10% of the final fluorescence.

### 4.3. Dynamic Light Scattering

The monomeric state of Aβ40 at the stock solution (250 μM, pH 11) was analyzed using a NANOTRAC FLEX in situ particle size analyzer (Microtrac TM). A total of 50 μL of the sample was added to the tip of the laser of the DLS equipment, and a 90 s analysis was performed.

### 4.4. Transmission Electron Microscopy

After 16 h of aggregation in low protein-binding microcentrifuge tubes, under the corresponding conditions, a 10 µL sample was placed onto carbon-coated copper grids, incubated for 5 min, and dried with Whatman paper. The grids were washed with distilled water, negatively stained with 2% (*w*/*v*) uranyl acetate for 2 min, and then dried. Micrographs were obtained using a JEM-1400 (JEOL, Tokyo, Japan) transmission electron microscope (TEM) operated at an accelerating voltage of 80 keV.

### 4.5. Infrared Spectroscopy

Infrared spectroscopy was performed as described by Benseny-Cases, 2007 [24]. Briefly, 100 μM peptide was incubated for 11, 15, and 24 h in sodium phosphate buffer in D_2_O at the corresponding pH at 37 °C under quiescent conditions. Then, 30 mL of peptide was deposited between two CaF2 windows separated by a 50 mm Teflon separator. All measurements were carried out on an FTIR Mattson Polaris spectrometer equipped with a liquid-nitrogen-cooled mercury–cadmium–telluride (MCT) detector at 37 °C. The spectrometer was purged continuously with dry air. For each spectrum, an average of one thousand scans were averaged at an instrumental resolution of 2 cm^−1^. The spectra were recorded in the range of 400 to 4000 cm^−1^. To obtain the infrared spectrum of the peptide, the spectrum of the solvent was subtracted from the sample, and all spectra were corrected for the atmospheric water vapor contribution. Each experiment was repeated three times.

### 4.6. Cell Cytotoxicity Assay

Neuroblastoma SH-SY5Y cells were cultured in Dulbecco’s modified Eagle’s medium (DMEM) supplemented with 10% (*v*/*v*) heat-inactivated fetal bovine serum, 1% glutamine, and 1% (*v*/*v*) penicillin/streptomycin. The cells were maintained at 37 °C and 5% CO_2_ in a 75 cm^2^ cell culture flask. Differentiation to neuronal cells started 24 h after plating by replacement of the maintenance medium with differentiation culture medium for 7 days and refreshment every 72 h. The differentiation culture medium consisted of DMEM supplemented with 2.5% inactivated FBS, 1% penicillin/streptomycin, 1% glutamine, and 10 μM retinoic acid. Differentiation was monitored microscopically by morphological assessment. 

For the cytotoxicity assay, the cells were seeded in 96-well plates and treated at a density of 2 × 10^4^ cells/well. Aβ40 at pH 5.5 and pH 7.4 was added at different concentrations, and after 24 h of incubation, cell viability was detected by MTT assay. After removing the medium, 10 μL of 3-(4,5-dimethylthiazol-2-yl)-2,5-diphenyltetrazolium bromide (MTT) solution (5 mg/mL) and 100μL of the medium were added to each well and incubated at 37 °C for 4 h. Then, 150 μL of dimethyl sulfoxide was added to each well to dissolve the formazan after discarding the supernatant. Absorbance values were quantified using a plate reader (TECAN Spark) at a wavelength of 490 nm. For each pH, the data are expressed as a percentage of viability with respect to non-treated cells. The non-treated cells were grown in a medium containing the same amount of buffer, at the corresponding pH, but without Aβ40. Figure S6 compares these two controls (pH 7.4 and pH 5.5) and a culture of cells non-treated and without the addition of any extra buffer (neither pH 7.4 nor pH 5.5).

## Data Availability

Not applicable.

## Supplementary Materials

The following supporting information can be downloaded at: https://www.mdpi.com/article/10.3390/ijms24098408/s1.
