# Supplementary material for: Aβ40 Aggregation under Changeable Conditions
Source: Int J Mol Sci. 2023 May 7;24(9):8408. doi: 10.3390/ijms24098408 (PMC10179685; doi:10.3390/ijms24098408)
Supplement: Supplementary file 1 [file ijms-24-08408-s001.zip › ijms-2356744-supplementary.pdf]

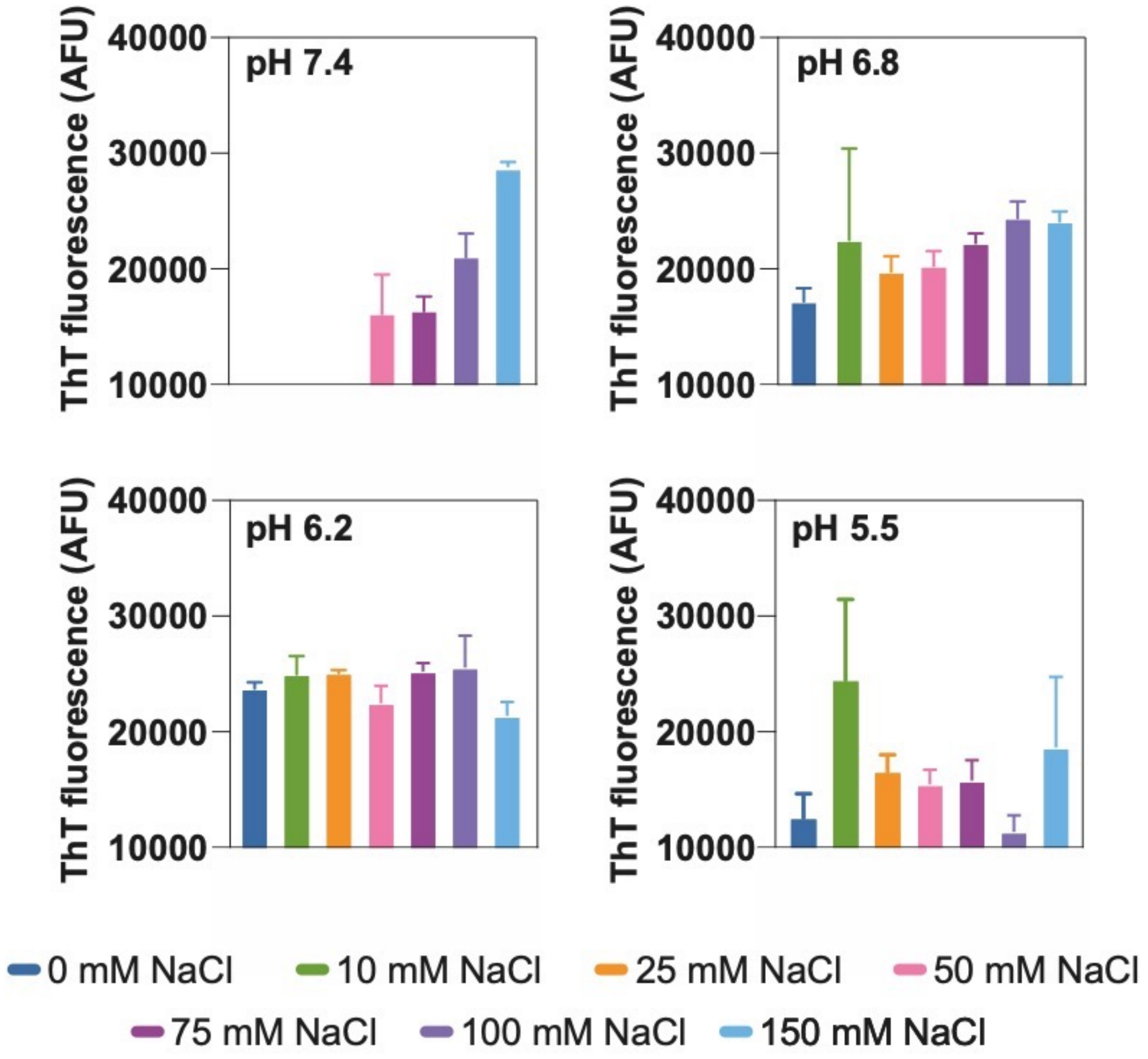

Figure S1. Plot showing the maximum Thioflavin-T signal of the kinetics presented in Figure 2 (panels A, B, C and D) against the NaCl concentration.

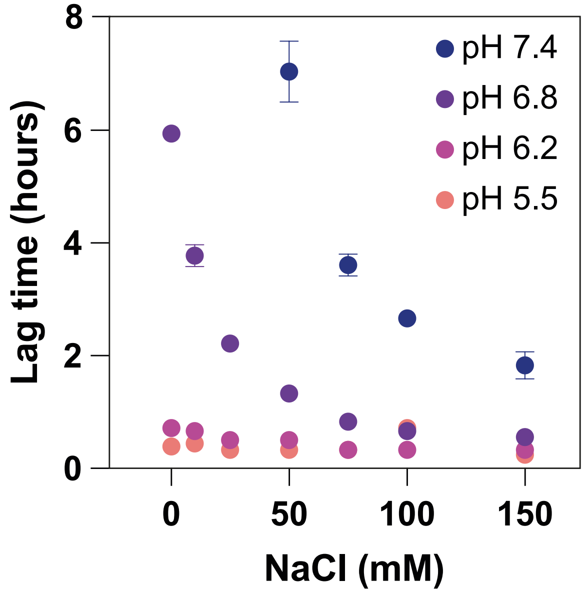

Figure S2. Plot showing the relationship between the lag-time of the kinetics presented in Figure 2 (panels A, B, C and D) against the NaCl concentration.

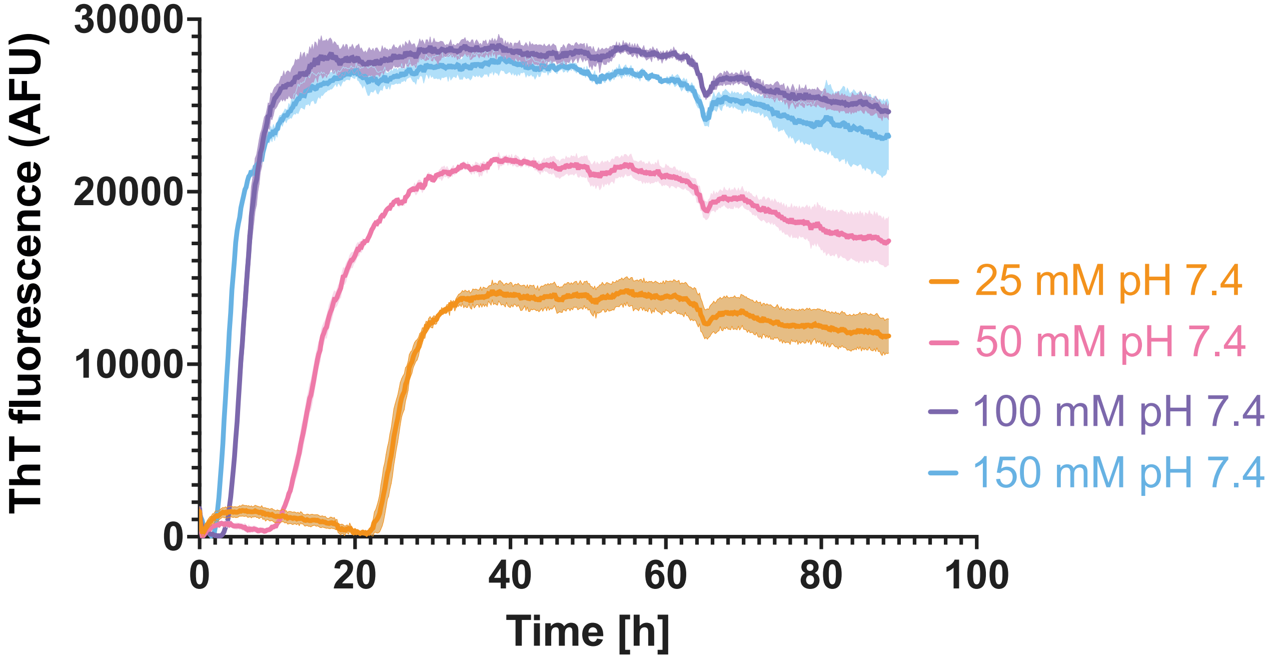

Figure S3. Plot showing reproducibility and stability of aggregation kinetics. Four examples at pH 7.4, of three replicates, showing a plateau above the 16 hours presented in Figure 1. This assay was performed on a different day and from different stock than those presented in Figure 1.

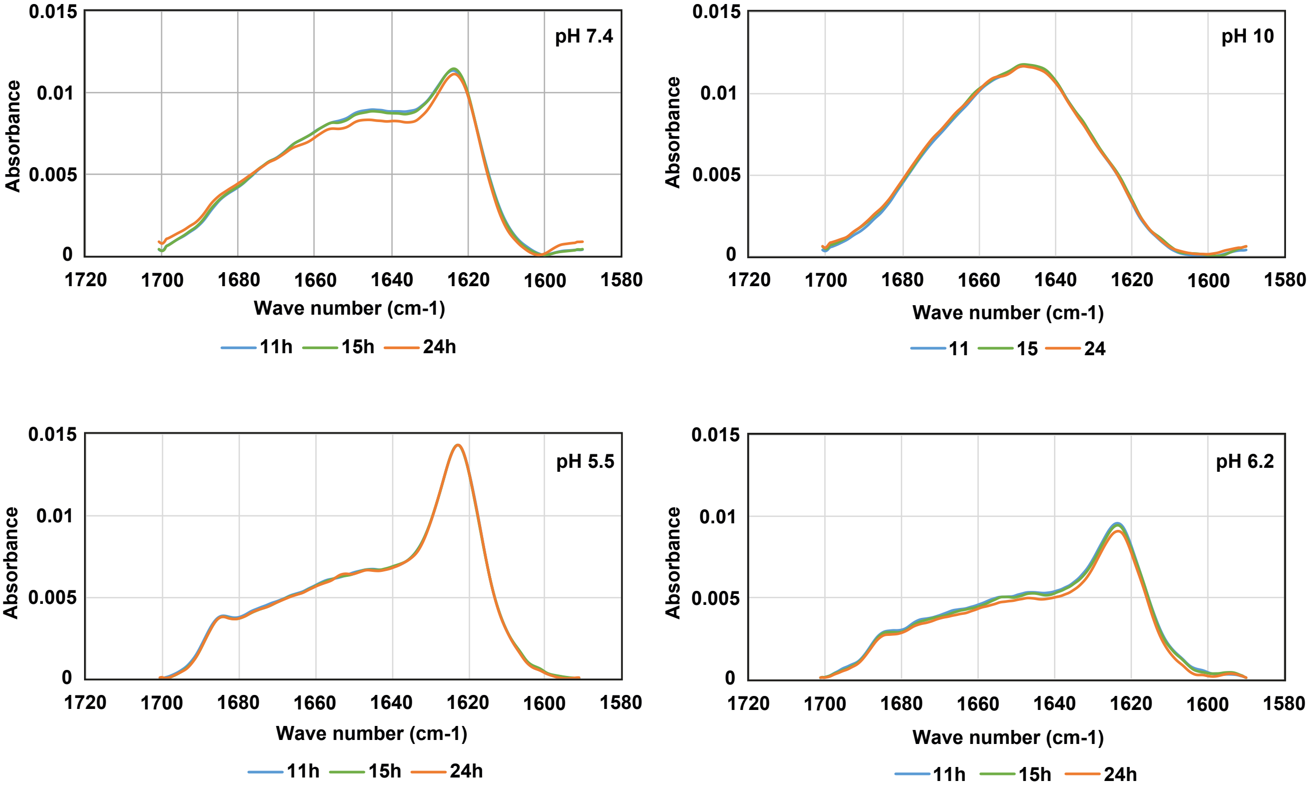

Figure S4. FTIR absorbance spectra of Aβ40 aggregates incubated at four different pHs (5.5, 6.2, 7.4 and 10) for 11, 15, and 24 hours. The absorbance remains stable from 11 to 24 hours, indicating a stable conformation during this period of time.

A

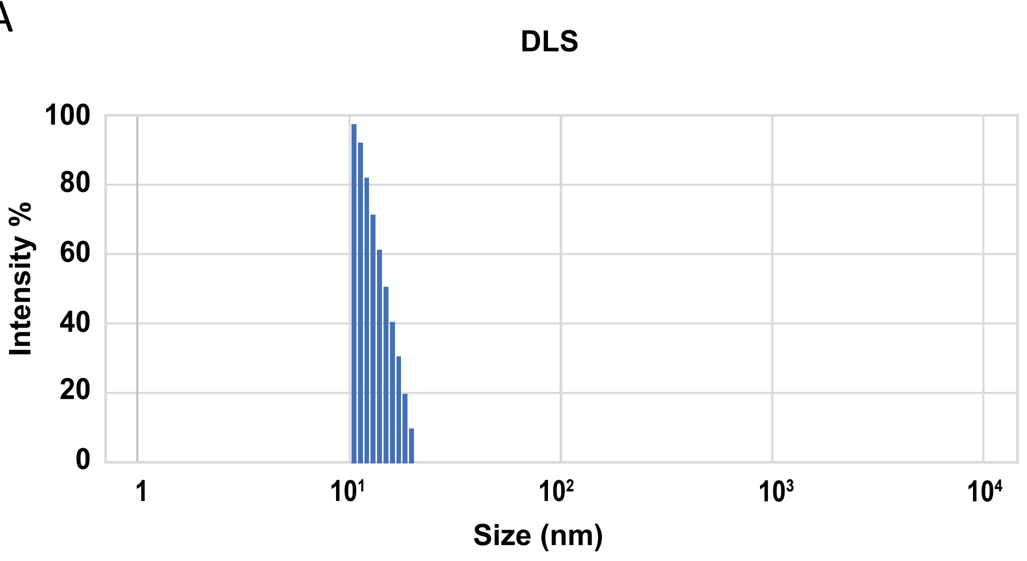

B

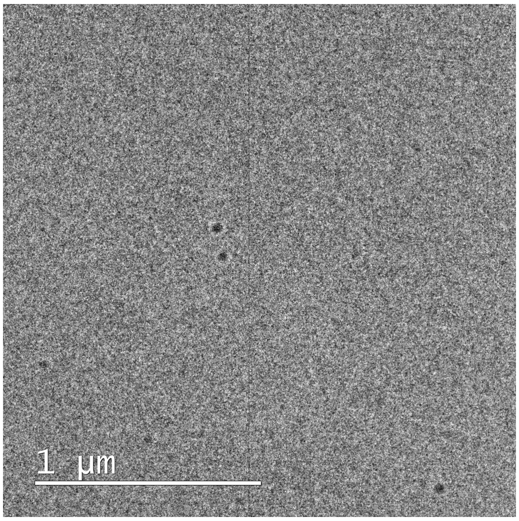

Figure S5. Absence of aggregates at the initial stock sample. A) Dynamic Light Scattering analysis of an initial stock sample (250 uM) of Aβ40 at pH 11. The signal shows just one particle size of 10 nanometers, which fits with the size of a monomeric Aβ peptide. B) TEM image for the initial stock sample (250 uM) of Aβ40 at pH 11.

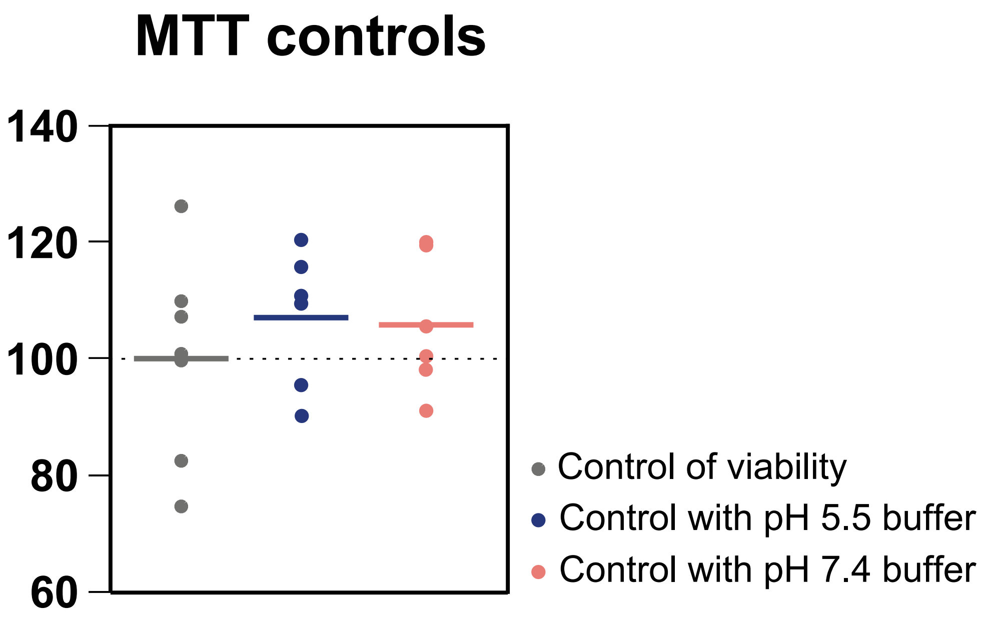

Figure S6. MTT viability assay. Percentage of cell viability, after incubating with buffers at pH 5.5 (blue) and pH 7.4 (red). The control of cells grown just in culture medium are shown in grey. The plot shows triplicates of two independent assays. The lines are the mean corresponding to each concentration of aggregates added. No significant difference was measured with Bonferroni test.
